# Continuous Versus Intermittent Linezolid Infusion for Critically Ill Patients with Hospital-Acquired and Ventilator-Associated Pneumonia: Efficacy and Safety Challenges

**DOI:** 10.3390/ph15030296

**Published:** 2022-02-28

**Authors:** Ahmed E. Abou Warda, Rania M. Sarhan, Hussein Saeed Al-Fishawy, Ayman N. Moharram, Heba F. Salem

**Affiliations:** 1 Clinical Pharmacy Department, Faculty of Pharmacy, October 6 University, Giza P.O. Box 12585, Egypt; ahmedessamabouwarda@gmail.com; 2 Clinical Pharmacy Department, Faculty of Pharmacy, Beni-Suef University, Beni-Suef P.O. Box 62514, Egypt; 3 Internal Medicine Department, Faculty of Medicine, Cairo University, Giza P.O. Box 12613, Egypt; hfishawe@hotmail.com; 4 Critical Care Medicine Department, Faculty of Medicine, Cairo University, Giza P.O. Box 12613, Egypt; ayman.moharram@kasralainy.edu.eg; 5 Pharmaceutics and Industrial Pharmacy Department, Faculty of Pharmacy, Beni-Suef University, Beni-Suef P.O. Box 62514, Egypt; heba_salem2004@yahoo.co.uk

**Keywords:** linezolid, intensive care unit, continuous infusion, intermittent infusion, clinical response, thrombocytopenia, pneumonia

## Abstract

High variability of linezolid blood concentrations with partial subtherapeutic levels was observed in critically ill patients who received a standard intravenous dose of linezolid, contributing to drug resistance and toxicity. Continuous infusions of linezolid have been suggested as an alternative and provide good serum and alveolar levels without fluctuations in trough concentration. This study aimed to assess the effectiveness and safety of continuous linezolid infusion versus the standard regimen in critically ill patients. A prospective randomized controlled study was conducted on 179 patients with nosocomial pneumonia. Patients were randomized into two groups. The first group received IV linezolid 600 mg twice daily, while the second group received 600 mg IV as a loading dose, followed by a continuous infusion of 1200 mg/day (50 mg/h) for at least 8–10 days. The continuous infusion group showed a higher clinical cure rate than the intermittent infusion group (*p* = 0.046). Furthermore, efficacy was proven by greater improvement of P/F ratio (*p* = 0.030) on day 7 of treatment, a lower incidence of developing sepsis after beginning treatment (*p* = 0.009), and a shorter time to reach clinical cure (*p* < 0.001). Hematological parameters were also assessed during the treatment to evaluate the safety between the two groups. The incidence of thrombocytopenia was significantly lower in the continuous infusion group than in the intermittent infusion group. In addition, a stepwise logistic regression model revealed that the intermittent infusion of linezolid was significantly associated with thrombocytopenia (OR =4.128; 95% CI = 1.681–10.139; *p* =0.001). The current study is the first to assess the clinical aspects of continuous infusion of linezolid beyond pharmacokinetic studies. Continuous infusion of linezolid outperforms intermittent delivery in safety and improves clinical effectiveness in critically ill patients with Gram-positive nosocomial pneumonia.

## 1. Introduction

Nosocomial pneumonia is the most common cause of mortality among intensive care unit (ICU) patients [1,2]. Despite advancements in antibiotics and adherence to guidelines, both hospital-acquired (HAP) and ventilator-associated pneumonia (VAP) can develop acute respiratory distress syndrome (ARDS) and acute lung injury (ALI), which result in high morbidity and mortality [3,4]. *Staphylococcus aureus* is one of the most causative Gram-positive bacteria in hospital-acquired and ventilator-acquired pneumonia, with mortality rates up to 40% [5,6].

Linezolid is effective against Gram-positive bacteria, including methicillin-resistant *Staphylococcus aureus* (MRSA), multidrug-resistant *Streptococcus pneumonia*, and vancomycin-resistant *Enterococci* (VRE) [7,8]. It is well established that linezolid has time-dependent activity, and the most critical pharmacokinetic parameters to predict its antibacterial effect include the time that the free drug concentrations remain above the minimum inhibitory concentration (T > MIC), as well as the area under the curve (AUC) over 24 h/minimum inhibitory concentration (MIC) (AUC/MIC) [9,10,11,12]. Recently, increased variability in linezolid blood concentrations associated with partial subtherapeutic serum drug levels has been reported in critically ill patients receiving linezolid’s usual dose (600 mg twice/day), which could lead to drug resistance and toxicity [13,14,15,16].

Continuous infusions of antibiotics have been proposed as an alternative to intermittent dosing to optimize the exposure period required to keep blood concentrations above MICs and permit the maximal effect of time-dependent antibiotics [14,17]. Based on T > MIC being a good linezolid efficacy predictor, it was observed that continuous linezolid infusion resulted in a higher T > MIC in critically ill patients. Moreover, it also provided good serum and alveolar levels with excellent alveolar diffusion and concentrations almost double the susceptibility breakpoint [18,19,20].

Linezolid is extensively safe and tolerated well, but its administration could be restricted by the risk of hematological adverse effects such as the development of anemia related to reduced hemoglobin levels and thrombocytopenia, defined as a platelet count of less than 1 × 10^5^/μL [21,22]. Thrombocytopenia is the most common adverse effect of linezolid treatment reported in several studies, and it is associated with a poor prognosis, a longer hospital stay, and mortality, especially in critically ill patients [23,24,25,26,27].

Several studies have demonstrated that the pharmacokinetic/pharmacodynamic target can be achieved when linezolid is used as a continuous infusion. However, evidence of its clinical outcomes is limited [19,20,28,29]. Thus, this study aimed to evaluate the efficacy and safety of continuous linezolid infusion versus the standard regimen in treating nosocomial pneumonia in the ICU.

## 2. Results

**Figure 1:**
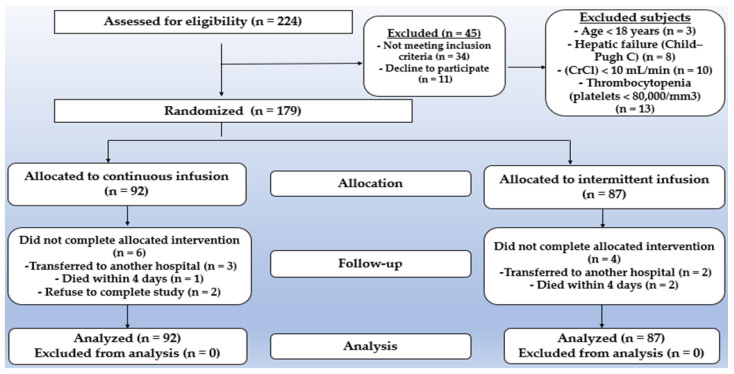
Flow chart of the final consort diagram displaying patient randomization.

During the research period, 224 patients were assessed for eligibility. After excluding 45 patients, 92 were randomly assigned to receive a continuous infusion and 87 received an intermittent infusion. Six patients from the continuous infusion and four patients from intermittent infusion were withdrawn from the trial due to death or transfer to another institution, but they were all included in the intention-to-treat analysis (Figure 1).

Using intention-to-treat (ITT) analysis, demographics and baseline characteristics in both groups were well matched. No significant differences were observed between the two groups except for creatinine clearance. The variables studied for each group are summarized in Table 1.

The clinical cure showed a significant difference between the two groups *(p* = 0.046), favoring continuous linezolid infusion (Table 2). In addition, the clinical cure was assessed for each bacterial strain in each group, as shown in Table 3.

The time to reach clinical cure was significantly lower (*p* < 0.001) in the continuous group. Besides, the duration of linezolid treatment showed a significant difference between the two groups (*p* = 0.037). Moreover, the number of patients who developed sepsis during treatment was lower in the continuous infusion group, with a significant difference between the two groups (*p* = 0.009). The length of hospital stay (LOS), ICU stays, mechanical ventilator (MV) use, mortality at the end of treatment, and 30-day mortality were not statistically lower in the continuous infusion group than in the intermittent group. Secondary outcomes are shown in Table 2.

Measures of hemoglobin, hematocrit, and platelet counts on days 1, 3, 5, 7, 9, 11, 13, and 15 during the treatment duration were compared between the patients in both groups. The results showed a decline in the hemoglobin (Hb) and hematocrit (Hct) levels during treatment from day 9 without any significant difference between the two groups on day 11 (*p* = 0.062), day 13 (*p* = 0.437), and day 15 (*p* = 0.931), and day 11 (*p* = 0.391), day 13 (*p* = 0.880), and day 15 (*p* = 0.484), respectively.

**Figure 2:**
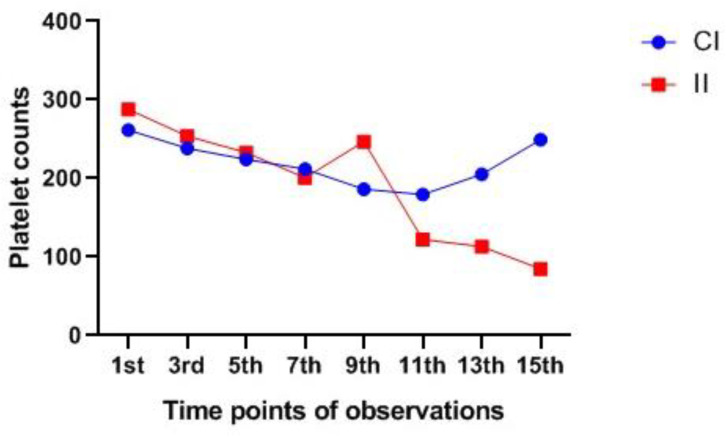
Comparison platelets count during linezolid treatment duration in each group. CI: continuous infusion, II: intermittent infusion.

The platelet count in the intermittent group appeared to be decreasing in comparison with patients in the continuous infusion group, with a significant difference between the two groups on day 11 (*p* = 0.001), day 13 (*p* = 0.005), and day 15 (*p* = 0.002) (Figure 2).

**Figure 3:**
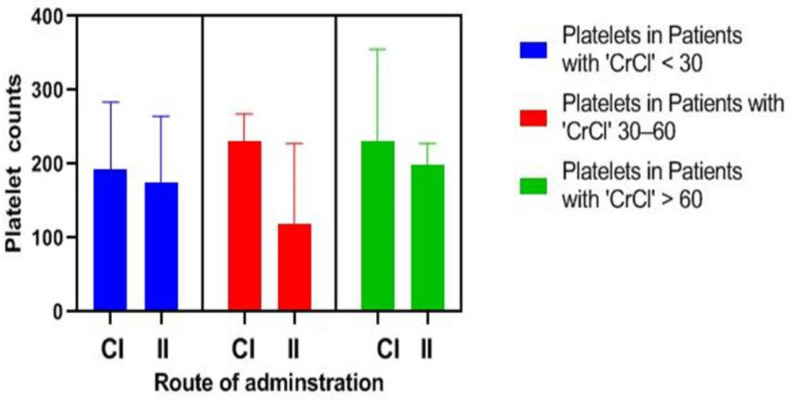
Comparison of platelet count during linezolid treatment duration according to different categories of creatinine clearance (CrCl) in each group. CI: continuous infusion, II: intermittent infusion.

Patients were stratified into three groups according to kidney function. As shown in Figure 3, patients with mild renal insufficiency (CrCl > 60) were significantly associated with a decreased risk of linezolid-induced thrombocytopenia in the continuous infusion (CI) group in comparison to the intermittent infusion (II) group on day 11 (*p* = 0.005), day 13 (*p* = 0.025), and day 15 (*p* = 0.016). Moreover, patients with moderate renal insufficiency (CrCl 30–60) were associated with a lower platelet count in the CI group than the II group on day 9 with a significant difference (*p* = 0.011). Concurrently, there was no significant difference between platelet count in the two groups in patients with severe renal insufficiency (CrCl < 30).

**Figure 4:**
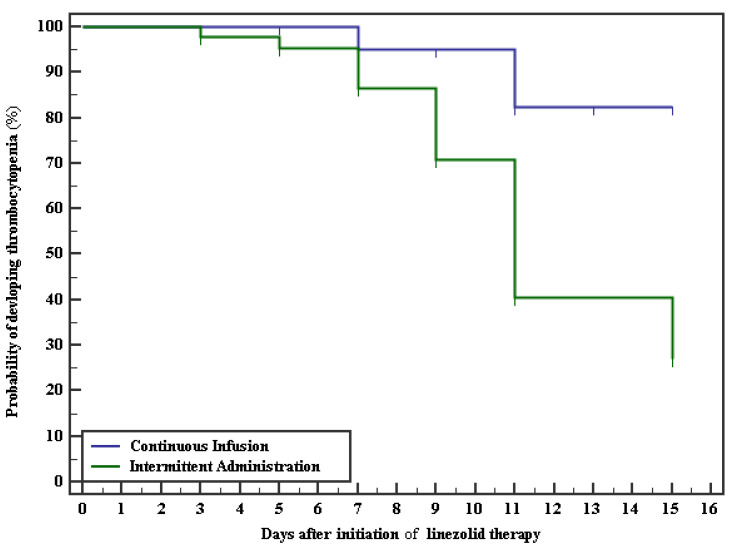
Kaplan–Meier curve shows the time from the initiation of linezolid therapy to the development of thrombocytopenia in the two groups, *p* < 0.001.

A survival analysis was conducted to evaluate the overall time from starting linezolid treatment to the occurrence of thrombocytopenia in both groups. The Kaplan–Meier curve and log-rank test showed a lower incidence of thrombocytopenia in the continuous infusion group (Figure 4).

A multivariate stepwise logistic regression model was used to predict parameters affecting clinical cure and risk factors for linezolid-induced thrombocytopenia, and the Hosmer–Lemeshow test was used to assess the fitness of model. Linezolid administration route (OR = 2.017, 95% CI = 1.088–3.738, *p* = 0.003), sepsis (OR = 3.037, 95% CI = 1.325–6.961, *p* = 0.008) and SOFA score (OR = 0.721, 95% CI = 0.612–0.849, *p* = 0.01) were the only independent predictors of clinical cure. The Hosmer–Lemeshow test results showed acceptable goodness of fit (*p* = 0.17). Table 4 shows the multivariate analysis for clinical cure predictors.

Indeed, as seen in Table 5, multivariate stepwise logistic regression showed that intermittent administration (OR = 4.128, 95% CI = 1.681–10.139, *p* = 0.001), baseline platelet count ≤ 200 × 10^3^/mm^3^ (OR = 3.148, 95% CI = 1.251–7.922, *p* =0.014) and CrCl < 30 mL/min (OR = 3.755, 95% CI = 3.755–8.664, *p* = 0.002) were considered significant risk factors for linezolid-related thrombocytopenia. The Hosmer–Lemeshow test results indicated a good fit model (*p* = 0.22).

## 3. Discussion

According to the current study, continuous infusion of linezolid achieved a significantly higher clinical cure rate and decreased the risk of development of linezolid-induced thrombocytopenia compared to intermittent administration in patients with hospital-acquired and ventilator-associated pneumonia. Regression analysis showed that continuous infusion was an independent predictor of clinical success, and intermittent infusion was considered a significant risk factor for linezolid-related thrombocytopenia. To the best of our knowledge, this is the first randomized controlled study that compared the clinical efficacy and safety of continuous infusion of linezolid versus intermittent administration in critically ill patients.

Linezolid has been frequently utilized over glycopeptides for the treatment of Gram-positive nosocomial pneumonia in critically ill patients following the increase in multidrug-resistant Gram-positive bacteria in the last decade, and glycopeptides have also been associated with frequent episodes of nephrotoxicity [9,30,31]. The most effective administration modality of parenteral antibiotics remains a controversial issue [32]. Recently, continuous administration of linezolid has been proposed as a valuable alternative to standard intermittent administration, especially in critically ill patients [28,33,34].

Several studies investigated continuous infusion of linezolid as a possible treatment option for critically ill patients with better pharmacokinetic/pharmacodynamic (PK/PD) targets. While all of these trials indicated that continuous infusion might be critical for maximizing the time above the MIC (T > MIC), very little information on the clinical effect was provided [18,19,20,35].

Our findings corroborated those of Adembri et al., who examined linezolid delivered intermittently or continuously to critically ill septic patients and monitored clinical outcomes. This study observed that continuous infusion had an advantage over intermittent infusion, and clinical cure was achieved in most patients, but without any significant differences in clinical success between the two groups. Nevertheless, the limitation of this study was the small sample size [20].

Another study by Boselli et al. reported that the administration of continuous infusion of linezolid might be superior over intermittent infusion in critically ill VAP patients providing T > MIC = 100%, even for strains with higher MICs [18]. Furthermore, Taubert et al. and Soraluce et al. found that the usual linezolid dosing was insufficient to achieve the therapeutic effect target and that continuous infusion was recommended [19,35]. However, those studies could not evaluate the clinical outcomes of continuous infusion of Linezolid. The present study compared the clinical efficacy between patients who received linezolid by continuous or intermittent infusion. It was shown that there was a statistically significant difference in the clinical cure rate between both groups. Moreover, efficacy was proven by a higher P/F ratio, a lower incidence of developing sepsis, and a shorter time to reach clinical cure in the continuous infusion group. Additionally, to take microbiological concerns into account, we assessed the patient’s cure for each bacterial isolate in both groups, and it was shown that the continuous linezolid treatment resulted in a more remarkable improvement in bacteriological efficacy than intermittent infusion.

Besides, even though there was a trend of higher clinical recovery in the continuous infusion group, there was no statistically significant difference in mortality rates between the groups; most likely as a result of patients who died from Gram-positive pneumonia developing sepsis, which was associated with persistent inflammation, immune suppression, and a hypercatabolic state, all of which resulted in poor outcomes [36,37]. Furthermore, most critically ill patients are at risk of death from other causes, such as cardiovascular disease or life-threatening arrhythmias, malignant failure, unexpected central nervous system deterioration, or sudden cardiac arrest [38].

On the other hand, the present study compared hematological adverse effects, especially thrombocytopenia. Our data revealed that the development of thrombocytopenia was significantly reduced in the continuous infusion group on days 11, 13, and 15. At the same time, anemia-associated linezolid therapy was similar throughout the treatment course between both groups. This was confirmed by several studies that reported the AUC_24_/MIC as one of the PK/PD parameters required for the efficacy and safety of linezolid [20,39,40,41]. It was observed that the AUC_24_/MIC target of 80–120 was achieved more constantly in the continuous infusion than in the intermittent administration [20]. Indeed, a recent study was conducted by Dou et al. to suggest that the occurrence of thrombocytopenia was only 23.5% when the AUC_24_/MIC ratio was between the target range [42]. In contrast, the possibility of thrombocytopenia was more than 50% when AUC24 > 400 mg h/L or exceeded the PK/PD target, following a 600 mg/12 h regimen [40,43]. Furthermore, a study suggested a dose of 500 mg/12 h due to the high occurrence of thrombocytopenia with the standard dosing regimen [44].

Recently, renal dysfunction has been considered one of the conditions related to thrombocytopenia in patients receiving linezolid [45]. Furthermore, patients with renal impairment (CLcr < 30 mL/min) had a higher risk of thrombocytopenia than patients with normal or mild kidney function (CLcr > 60 mL/min) [46,47]. The current study compared platelet counts in each group between patients with normal or mild renal dysfunction and those with moderate and severe renal insufficiency. It was found that the incidence rate of linezolid-induced thrombocytopenia was significantly lower only in patients administered linezolid by continuous infusion (CLcr > 30 mL/min).

Several studies evaluated the risk factors associated with the occurrence of linezolid-induced thrombocytopenia [48,49,50,51]. Niwa et al. [52] determined the extent of thrombocytopenia in patients with a low baseline platelet count. Zhang et al. [53] reported that the thrombocytopenia in patients receiving linezolid had a significant increase among patients aged ≥ 65 years. Hanai et al. [54] considered that patients who received linezolid for more than 14 days were at an increased risk of becoming thrombocytopenic, and their platelet count should be closely monitored.

The current study revealed that the intermittent infusion of linezolid in addition to baseline platelets ≤ 200 × 10^3^/mm^3^ and with CrCl < 30 mL/min was considered as a risk factor for linezolid-induced thrombocytopenia in critically ill patients. Moreover, it should be mentioned that the longer duration (>14 days) might not be shown significantly due to the limited number of patients that exceeded 14-day treatment in this study.

Some limitations should be considered. First, the study design would have been enhanced by a double-blind, double-dummy approach. However, tested parameters were objective to overcome this limitation. Second, we did not investigate the microbiological cure and bacterial eradication at the end of the linezolid treatment, as despite clinical cures, bacteria might persist, particularly in chronic obstructive pulmonary disease [55], and new pathogens may colonize in the oral cavity, leading to false-positive results [56]. In addition, it was challenging to obtain a valid sample after treatment. Third, the Cockcroft–Gault equation was used to estimate creatinine clearance as it was more frequently implemented in the clinical setting, but the accuracy of this equation was biased by body weight and body mass index, which could lead to overestimation of creatinine clearance compared to the MDRD or CKD EPI equation [57,58]. Finally, limited financial resources affected the measuring of patients’ linezolid concentrations.

Despite these limitations, the findings of this study may stimulate interest in research on continuous infusion usage of linezolid. We focused on the clinical efficacy of linezolid delivered via continuous infusion in treating Gram-positive bacterial pneumonia. However, our findings may not be generalizable to other infection sites or patients who are not critically ill.

## 4. Materials and Methods

### 4.1. Study Design and Setting

This prospective randomized controlled study was conducted between November 2019 and July 2021 at the ICU of the Critical Care Department, Cairo University Hospitals, Cairo, Egypt. The study protocol was registered at clinicaltrials.gov (Registration number: NCT04531332).

The study was approved by the Ethics Committee and Institutional Review Boards of the Faculty of Pharmacy, Beni-Suef University, and the Critical Care Medicine Department, Cairo University (Approval Number: REC-H-PhBSU-20004). The study was conducted as per the principles outlined in the Declaration of Helsinki [59]. Written informed consent was obtained from all participants or their legal guardians.

### 4.2. Patients

The inclusion criteria included adults aged > 18 years with clinical suspicion of HAP (new persistent radiological infiltrate after more than 48 h of hospital admission with two of the following signs: fever ≥ 38 °C, purulent tracheal aspirations, or white blood cells count (WBCs) > 10,000/mL). With the criteria above, patients who were mechanically ventilated for more than 48 h, were classified as VAP [60]. Positive culture confirming linezolid susceptibility was essential for inclusion.

Exclusion criteria included pregnancy or lactation, allergy to linezolid, baseline creatinine clearance (CrCl) < 10 mL/min calculated directly using the Cockcroft–Gault equation based on the serum creatinine value, severe hepatic failure (Child-Pugh C), thrombocytopenia (platelet count < 80,000/mm^3^), disseminated intravascular coagulation (DIC), hematologic disease, and concurrent use of other medications that may interact with linezolid (i.e., macrolides, serotonin modulators), or drug-associated thrombocytopenia.

### 4.3. Patient’s Randomization and Protocol

Initially, each patient was evaluated for HAP or VAP based on the clinical criteria. Baseline microbiologic specimens were obtained for all recruited patients through the day of diagnosis. Acceptable semi-quantitative non-invasive methods comprised an assembly of normally expectorated samples, nasotracheal suctioning or endotracheal aspiration, and sputum production for Gram’s stain and culture. Then, empirical treatment with meropenem 1 g IV every eight hours was maintained until culture results [61]. If Gram-negative or mixed Gram-positive and Gram-negative pathogens were identified, the patient would be excluded from the study. Patients with Gram-positive cultures sensitive to Linezolid were randomly allocated to receive Linezolid in the continuous infusion group or conventional intermittent infusion group in a 1:1 ratio. Patients in the continuous infusion group received an intravenous loading dose of 600 mg for 60 min, followed by a continuous infusion of 1200 mg/day (50 mg/h). The intermittent infusion group administered 600 mg of linezolid IV every 12 h for 60 min.

The treatment period was 8–10 days in both groups but might decrease or increase based on the patients’ clinical response. At the beginning of the study, simple randomization was used to assign patients using the random numbers set offered by the ICU coordinator. Each patient was assigned a randomized code consisting of two letters that indicate the intensive care unit and two digits indicating the randomization sequence.

### 4.4. Patient Follow-Up and Outcome Measures

Daily clinical response was evaluated using signs and symptoms, sputum purulence, fever, X-ray, and total leukocyte count. On entry to the study, predicted mortality rates in the two groups were computed using the Sequential Organ Failure Assessment (SOFA) score and the Simplified Acute Physiology II Score (SAPS II).

The primary efficacy outcome was defined as a clinical cure on day 7 following linezolid initiation. Clinical cure was indicated as normal body temperature, TLC < 10,000/mL [62], improvement in oxygenation with Pao2/Fio2 > 250, and no requirement for oxygen supplementation [63], hemodynamic stability, resolution of radiographic pulmonary infiltrate [64,65], and absence of purulent secretions [66].

The secondary efficacy outcomes included the duration of hospital stay, ICU stay, linezolid use, mechanical ventilator use, MV-free days, and treatment until clinical cure. The proportion of patients in each group that developed sepsis was defined as a patient’s SOFA score of 2 or more after diagnosis with HAP or VAP and at the beginning of treatment regimen, and mortality at the end of treatment, and 30-day mortality were also included as secondary outcomes. Additionally, the patient’s clinical cure for each microbiological isolate was examined in each group.

Furthermore, to evaluate the safety between the two groups, the changes in the patients’ hematological parameters (hemoglobin (Hb), hematocrit (Hct), and platelet (PLT) count were assessed every two days during linezolid treatment. The relationship between kidney function and the development of linezolid-induced thrombocytopenia was also investigated. The patients were categorized into three groups based on their creatinine clearance CrCL (mL/min), calculated using the Cockcroft–Gault formula. The CrCL ranges of the three groups were as follows: CrCl > 60 mL/min (mild kidney impairment), CrCl 30–60 mL/min (moderate kidney impairment), and CrCl < 30 mL/min (severe kidney impairment). The platelet counts were compared between each group to assess the incidence of linezolid-associated thrombocytopenia among different renal functions. Thrombocytopenia was defined as a decrease in platelet count ≥ 50% from baseline or a decrease in platelet count ≤ 100 × 10^3^/mm^3^ [67].

### 4.5. Statistical Analysis

The sample size was calculated based on an expected 25% absolute difference in the clinical cure rate. The total sample size calculated was 169 patients. Alpha and power were kept at 0.05 and 0.9, respectively. The sample size was increased to 179 patients to compensate for a predicted loss of 5% to 10%.

Data were analyzed using IBM SPSS software version 20.0 (IBM Corp.: Armonk, NY, USA). The Kolmogorov–Smirnov test was used to check the normality of variables. Categorical variables were compared using the chi-square or Fisher’s exact test. An independent t-test and a Mann–Whitney U test were used to compare the two groups for normally and non-normally distributed quantitative variables, respectively. Multivariate stepwise logistic regression was used to detect clinical cure predictors and investigate the risk factors for thrombocytopenia. All independent variables were evaluated separately in the univariate analysis model. Moreover, variables with a *p* < 0.1 in the univariate analysis were chosen for further multivariate regression using the Akaike information criterion (AIC). A Hosmer–Lemeshow goodness of fit test was used to assess the performance of the analysis. The Kaplan–Meier survival curve was used to meet safety endpoints with a log-rank test to interpret the significant difference in median time. All statistical tests were two-tailed and considered significant at a *p*-value < 0.05.

## 5. Conclusions

As far as we know, this is the first study to assess the clinical aspects, efficacy, safety, and significance of continuous infusion of linezolid compared to intermittent infusion. Besides, our study can stimulate interest and allow clinical research into the continuous infusion of linezolid beyond pharmacokinetic studies. Continuous infusion of linezolid outperforms intermittent delivery in terms of safety because it reduces the development of thrombocytopenia and improves clinical effectiveness in critically ill patients with Gram-positive nosocomial pneumonia. Further studies are recommended to illustrate the clinical efficacy of continuous infusion.

## Figures and Tables

**Table 1:** Baseline and demographic data in both groups.

Parameters	CI Group (*n* = 92)	II Group (*n* = 87)	*p*-Value
**Gender, *n*%**			
Male	58 (63.0%)	55 (63.2%)	0.981
Female	34 (37.0%)	32 (36.8%)	
**Age (years)**			
Min.–Max.	42.0–88.0	29.0–88.0	0.865
Mean ± SD	66.55 ± 10.56	66.89 ± 14.93	
**Body Mass Index** **(BMI) (kg/m^2^)**			
Min.–Max.	20.20–53.60	22.0–49.10	0.190
Mean ± SD	30.24 ± 6.97	28.99 ± 5.58	
**(SAPS) II**			
Min.–Max.	31.0–69.0	25.0–66.0	0.195
Mean ± SD	43.80 ± 8.61	42.10 ± 8.86	
**SOFA**			0.579
Min.–Max.	3.0–11.0	3.0–13.0	
Median (IQR)	5.0 (4.0–7.0)	5.0 (3.50–7.0)	
**Type of pneumonia, *n*%**			
HAP	58 (63.0%)	59 (67.8%)	0.502
VAP	34 (37.0%)	28 (32.2%)	
**CPIS for VAP.**			
Min.–Max	6.0–9.0	6.0–9.0	0.941
Mean ± SD	7.26 ± 0.83	7.25 ± 0.70	
**HAP patients required mechanical ventilation, *n*%**	13 (22.4%)	16 (27.1%)	0.669
**WBCs count** **(>11,000 cells/mm)**			
Min.–Max.	8.30–33.90	5.53–42.30	0.959
Median (IQR)	13.15 (11.50–21.0)	14.20 (11.10–20.20)	
**Body temperature** **(>38 °C), *n*%**	30 (32.6%)	30 (34.5%)	0.791
**Baseline CRP (mg/dL)**			
Min.–Max.	3.30–282.20	3.0–200.0	0.786
Median (IQR)	77.0 (17.30–161.0)	75.0 (35.50–134.0)	
**Baseline PCT (ng/mL)**			
Min.–Max.	0.60–37.0	0.29–27.0	0.709
Median (IQR)	2.90 (1.90–9.80)	3.40 (1.61–7.90)	
**Baseline(P/F) ratio**			
Min.–Max.	77.10–298.0	62.80–315.0	0.072
Median (IQR)	151.0 (110.0–190.0)	174.0 (119.0–254.5)	
**Baseline serum creatinine(S.Cr) (mg/dL)**			
Min.–Max.	0.50–4.10	0.40–7.30	<0.001 *
Median (IQR)	1.21 (0.80–2.20)	2.50 (1.02–3.96)	
**Baseline creatinine clearance (CrCl) (mL/min)**			
Min.–Max.	13.0–136.0	11.0–122.0	<0.001 *
Median (IQR)	53.05 (28.0–80.0)	28.39 (18.73–64.0)	
**Radiological findings**			
**Multi lobar infiltrates, *n*%**	54 (58.7%)	49 (56.3%)	0.748
**Pleural effusion, *n*%**	36 (39.1%)	44 (50.6%)	0.124

IQR: interquartile range; SD: standard deviation; *: statistically significant. (SAPS) II: Simplified Acute Physiology Score II, SOFA: Sequential Organ Failure Assessment Score, CPIS: Clinical Pulmonary Infection Score, VAP: Ventilator-associated pneumonia, HAP: Hospital acquired pneumonia, WBCs: white blood cells, CRP: C-Reactive protein, PCT: Procalcitonin, P/F: PaO2/FIO2

**Table 2:** Clinical outcomes in both groups after linezolid administration.

Parameters	CI Group (*n* = 92)	II Group (*n* = 87)	*p*-Value
**Clinical cure, *n*%**	56 (60.9%)	40 (46.0%)	0.046 *
**Development of sepsis, *n*%**	26 (28.3%)	41 (47.1%)	0.009 *
**Mortality at the end of linezolid treatment, *n*%**	10 (10.9%)	13 (14.9%)	0.510
**30-Day mortality, *n*%**	26 (28.3%)	19 (21.8%)	0.322
**P/F ratio at the seventh day of treatment**			
Min.–Max.	79.0–429.0	71.0–370.0	0.030 *
Median (IQR)	246.0 (181.0–312.0)	198.0 (122.5–275.5)	
**Length of ICU stay**			
Min.–Max.	3.0–24.0	3.0–27.0	0.188
Median (IQR)	9.0 (7.0–11.0)	10.0 (7.0–12.0)	
**Length of hospital stay**			
Min.–Max.	3.0–26.0	3.0–30.0	0.063
Median (IQR)	11.0 (9.50–13.0)	12.0 (9.50–14.0)	
**Days of treatment on Linezolid**			
Min.–Max.	3.0–14.0	3.0–14.0	0.037 *
Median (IQR)	7.0 (7.0–11.0)	7.0 (7.0–9.0)	
**Days to reach the clinical cure**			
Min.–Max.	4.0–7.0	5.0–10.0	<0.001 *
Mean ± SD	5.82 ± 1.21	6.96 ± 0.97	
**Duration on Mechanical ventillation (MV)**			
Min.–Max.	2.0–11.0	2.0–14.0	0.422
Median (IQR)	4.0 (3.0–6.0)	5.0 (3.0–7.0)	
**(MV) free days**			
Min.–Max.	2.0–20.0	2.0–19.0	0.194
Median (IQR)	6.0 (4.0–8.0)	5.0 (3.0–8.0)	

IQR: interquartile range; SD: standard deviation; *: statistically significant.

**Table 3:** Microbiological data and clinical cure according to bacterial isolate.

Parameters	*Streptococcus pneumoniae*	Methicillin-Resistant *Staphylococcus aureus* (MRSA)	Methicillin-Susceptible *Staphylococcus aureus* (MSSA)
No. (%) of patients with VAP	11 (17.7%)	43 (69.4%)	8 (12.9%)
No. (%) of patients with HAP	15 (12.8%)	58 (49.6%)	44 (37.6%)
No. (%) of cured patients in CI group	8 (14.3%)	30 (53.6%)	18 (32.1%)
No. (%) of cured patients in II group	9 (22.5%)	17 (42.5%)	14 (35.0%)

**Table 4:** Multivariate stepwise logistic regression analysis for parameters affecting a clinical cure.

Parameters	OR	95% CI	*p* Value
**Route of linezolid administration (continuous infusion)**	2.017	1.088–3.738	0.003 *
**Development of sepsis**	3.037	1.325–6.961	0.008 *
**SOFA score**	0.721	0.612–0.849	0.01 *

Akaike information criterion (AIC) = 202.91. OR: odds ratio, CI: confidence interval, *: statistically significant.

**Table 5:** Multivariate stepwise logistic regression analysis for risk factors involving the incidence of linezolid-induced thrombocytopenia.

Parameters	OR	95% CI	*p*-Value
**Route of linezolid administration (intermittent infusion)**	4.128	1.681–10.139	0.001 *
**Baseline platelets < 200 × 10^3^/mm^3^**	3.148	1.251–7.922	0.014 *
**CrCl < 30**	3.755	3.755–8.664	0.002 *

Akaike information criterion (AIC) = 142.06. OR: odds ratio, CI: confidence interval, *: statistically significant.

## Data Availability

Data is contained in the article.
